# Neoadjuvant PD-1 blockade combined with chemotherapy is not superior to neoadjuvant chemotherapy alone in resectable locally advanced esophageal carcinoma

**DOI:** 10.1186/s12957-023-02915-z

**Published:** 2023-02-03

**Authors:** Daoan Cheng, Weiqing Zhao, Rui Chen, Dong Li, Shuxian Tang, Cheng Fang, Mei Ji

**Affiliations:** Departments of Oncology, the Third Affiliated Hospital of Soochow University, Changzhou, 213004 China

**Keywords:** Anti-programmed death-1 (PD-1), Esophageal cancer, Neoadjuvant treatment, Immunochemotherapy

## Abstract

**Background:**

Neoadjuvant chemotherapy (nCT) or neoadjuvant chemoradiotherapy followed by surgery has been recommended as standard treatment in patients with locally advanced esophageal cancer (LAEC). But the risk of tumor recurrence still remained, and many patients refused or abandoned radiotherapy because of the intolerable adverse effects in China. Neoadjuvant immunochemotherapy (nICT) followed by surgery has become an emerging treatment in patients with esophageal cancer. There was still no consensus on whether nICT was superior to nCT alone in patients with esophageal cancer.

**Methods:**

In this retrospective study, patients with resectable esophageal cancer who received surgery after nICT (*n*=26, 40%) or nCT alone (*n*=39, 60%) were included. The patients were classified as nICT or nCT arm. The primary endpoints were pathological tumor response (PTR) and event-free survival (EFS). The different clinic-pathological features were compared by the Kruskal-Wallis test for continuous variables and the Chi-square (*χ*^2^) test for categorical variables. Kaplan-Meier curves were used to estimate EFS from the date of treatment to recurrence or death. All tests were 2-sided with a significative *P*-value defined <.05.

**Results:**

Three (11.5%) of the 26 patients achieved pathological complete remission (pCR) in the nICT group, and four (10.3%) of the 39 patients achieved pCR in the nCT group, respectively (*P*=1.000). Six (23.1%) of the 26 patients achieved major pathological response (MPR) in the nICT group, and 11 (28.2%) of the 39 patients achieved MPR in the nCT group, respectively (*P*=0.645). Downstaging was achieved in 13 (44.8%) patients in the nICT group and 16 (55.2%) patients in the nCT group, respectively (*P*=0.732). To verify the tumor regression grade (TRG) results, we compared them with MPR and pCR, which showed a significant dependency (*P*< 0.001). Patients who achieved downgrading showed better MPR and pCR rates (*P*<0.001 and *P* =0.010). There was no significant difference in EFS between the nICT and nCT groups (HR=1.011, 95% CI: 0.421–2.425, *P* = 0.981).

**Conclusions:**

Neoadjuvant PD-1 blockade combined with chemotherapy was not superior to chemotherapy alone for patients with resectable locally advanced esophageal carcinoma. However, more studies with long-term follow-up were needed to confirm this result.

## Background

Globally, cancer remains a significant health issue for humanity, with esophageal cancer being the sixth leading cause of cancer-related deaths [1]. The 5-year overall survival rate of patients with esophageal cancer was between 12 and 20%, and esophageal cancer treatments faced enormous challenges [2, 3].

Neoadjuvant chemotherapy (nCT) or neoadjuvant chemoradiotherapy (nCRT) before surgery had a better prognosis than those who received surgery alone for patients with locally advanced esophageal cancer (LAEC) [4]. Based on a large number of clinical evidence [4–8], nCT or nCRT has been recommended as standard treatment in patients with LAEC [9]. However, although the prognosis of patients was improved to some extent, patients still faced the risk of cancer recurrence [9]. A multicentre Phase III trial (NEOCRTEC5010) showed that the 5-year cumulative total recurrence rate of patients with locally advanced esophageal squamous cell carcinoma (ESCC) treated with nCRT was 32.2%, the local recurrence rate was 15.3%, and the distant recurrence rate was 24.3%, respectively [7, 9]. On the other hand, a meta-analysis demonstrated that nCRT had a higher 3-year survival benefit than nCT including local recurrence rate and distant metastasis rate; however, there was no increase in 5-year survival [10]. Radiotherapy was limited by patient compliance, and many patients refused or abandoned radiotherapy because of the intolerable adverse effects in China [11]. Therefore, developing a new treatment model was necessary to improve the prognosis of patients with LAEC.

Recently, neoadjuvant immunochemotherapy (nICT), such as camrelizumab, sintilimab, or tislelizumab combined with chemotherapy, has shown acceptable safety and high efficacy in patients with LAEC [12–14]. In addition, Xu et al. demonstrated that nICT and nCRT had comparable R0 resection rates (90.9% vs. 89.0%, *P*=0.302) and pathological complete remission (pCR) rate (29.8% vs. 34.0%, *P*=0.167) in ESCC [15]. Of note, there was still no clear evidence of the prognostic difference between nCT and nICT. More clinical evidence was needed to support the feasibility of nICT.

Several studies have found that pathological tumor response (PTR), such as tumor regression grade (TRG) and downstaging, was a prognostic factor for patients with esophageal cancer who received neoadjuvant treatment [16–19]. This study compared the differences in pCR rate, major pathological response (MPR) rate, TRG, downstaging, and event-free survival (EFS) between nICT and nCT groups to investigate whether neoadjuvant immunochemotherapy was superior to chemotherapy alone in patients with esophageal cancer.

## Materials and methods

### Patients

This retrospective study enrolled 65 patients with stage II to III esophageal cancer who received esophagectomy after neoadjuvant immunochemotherapy or chemotherapy. All patients were diagnosed with esophageal cancer by gastroscopic biopsy before surgery.

### Study design

The patients were classified as receiving nICT or nCT arm. TRG grading system was designed to classify regressive changes after neoadjuvant treatment based on histopathological results to reveal prognostic information. The College of American Pathologists (CAP) grading system was used to assess tumor response. We classified the degree of degeneration of histomorphology into the following four categories: grade 0, no residual cancer cells (pCR); grade 1, single cells or small groups of cancer cells; grade 2, residual cancer with evident tumor regression but more than single cells or rare small groups of cancer cells; and grade 3, extensive residual cancer with no evident tumor regression. Pathological regression was assessed using hematoxylin and eosin (H&E) stained slides of surgical specimens. Two independent radiologists or pathologists reviewed all imaging data and pathological data. Tumors with ≤10% residual viable tumor cells were considered as having achieved an MPR, while those showing no viable residual tumor were defined as achieving a pCR. Patients with ≥50% residual viable tumors were defined as non-responders. The eighth AJCC criteria were used for esophageal cancer staging. Regular follow-up, including computed tomography (CT) scans to monitor for recurrence of the disease. Adverse events were continually monitored throughout the study from the time of the beginning of treatment to 30 days after the surgery. Adverse events were categorized according to the Medical Dictionary for Regulatory Activities and assessed as per the National Cancer Institute Common Terminology Criteria for Adverse Events (version 5.0).

### Statistical analysis

Statistical analyses were performed using SPSS 17.0 (SPSS Inc, Chicago, IL, USA) software and presented with the GraphPad Prism software (GraphPad Software, San Diego, CA, USA). Continuous data were expressed as median with the range. Classified variables were shown as counts and percentages. The different clinic-pathological features were compared by the Kruskal-Wallis test for continuous variables and the chi-square (*χ*^2^) test for categorical variables. Kaplan-Meier curves were used to estimate EFS from the date of treatment to recurrence or death. A two-sided *P* value <0.05 was considered statistically significant.

## Results

### Baseline characteristics

This study enrolled 65 patients, including 54 (83.1%) males and 11 (16.9%) females. The median age was 67 years (range: 44–79). According to endoscopic diagnosis, 2 (3.1%) tumors occurred in the proximal esophagus, 24 (36.9%) in the middle esophagus, 34 (52.3%) in the distal esophagus, and 5 (7.7%) in the gastroesophageal junction. By pathological biopsy, 57 (87.7%) patients were diagnosed with esophageal squamous carcinoma, 5 (7.7%) with esophageal adenocarcinoma, and 3 (4.6%) with other pathological types. 33 (50.8%) and 32 (49.2%) were TNM stages II and III before treatment of esophageal carcinoma. The median cycle of treatment was 2 (range, 1–6). Preoperative treatment: 15 patients (23.1%) received sintilimab in combination with platinum and paclitaxel chemotherapy. Seven patients (10.8%) received camrelizumab combined with platinum and paclitaxel chemotherapy. Three patients (4.6%) received tislelizumab combined with platinum and paclitaxel chemotherapy. And 1 patient (1.5%) received pembrolizumab combined with platinum and paclitaxel chemotherapy. Thirty-nine patients (60%) received platinum combined with paclitaxel chemotherapy. Postoperative adjuvant therapy: In the nICT group, 7 patients did not receive adjuvant therapy after surgery, 14 patients received immunotherapy combined with chemotherapy after surgery, 4 patients received radiotherapy after surgery, and 1 patient received chemotherapy after surgery. In the nCT group, 19 patients did not receive adjuvant therapy after surgery, 13 received chemotherapy after surgery, and 7 patients received radiotherapy after surgery. Patients’ characteristics were summarized in Table 1.

**Table 1:** Clinical characteristics of the 65 patients in this study

Characteristic	Category	All patients (*n*=65)	nICT (*n*=26)	nCT (*n*=39)	*P*
Age (years)	Median (range)	67 (44–79)	67 (44–79)	67 (48–73)	
Sex					1.000
	Male	54 (83.1%)	22 (40.7%)	32 (59.3%)	
	Female	11 (16.9%)	4 (36.4%)	7 (63.6%)	
Location					0.199
	Proximal	2 (3.1%)	1 (50.0%)	1 (50.0%)	
	Middle	24 (36.9%)	9 (37.5%)	15 (62.5%)	
	Distal	34 (52.3%)	16 (47.1%)	18 (52.9%)	
	Gastroesophageal junction	5 (7.7%)	0 (0.0%)	5 (100.0%)	
Histology					0.167
	Squamous cell carcinoma	57 (87.7%)	25 (43.9%)	32 (56.1%)	
	Adenocarcinoma	5 (7.7%)	0 (0.0%)	5 (100.0%)	
	Others	3 (4.6%)	1 (33.3%)	2 (66.7%)	
c-TNM					0.685
	II	33 (50.8%)	14 (42.4%)	19 (57.6%)	
	III	32 (49.2%)	12 (37.5%)	20 (62.5%)	
Adjuvant therapy					0.079
	Yes	39 (60.0%)	19 (48.7%)	20 (51.3%)	
	No	26 (40.0%)	7 (26.9%)	19 (73.1%)	
Adjuvant chemotherapy					0.005
	Yes	14 (21.5%)	1 (7.1%)	13 (92.9%)	
	No	51 (78.5%)	25 (49.0%)	26 (51.0%)	
Adjuvant immunochemotherapy				<0.001	
	Yes	14 (21.5%)	14 (100%)	0 (0.0%)	
	No	51 (78.5%)	12 (23.5%)	39 (76.5%)	
Adjuvant radiotherapy					1.000
	Yes	11 (16.9%)	4 (36.4%)	7 (63.6%)	
	No	54 (83.1%)	22 (40.7%)	32 (59.3%)	

*nICT* neoadjuvant immunochemotherapy, *nCT* neoadjuvant chemotherapy

### Pathological tumor response (PTR)

TRG grading was performed on postoperative specimens from 65 patients, including 26 in the nICT group (grade 0, *n*=3, 11.5%; grade 1, *n*=3, 11.5%; grade 2, *n*=8, 30.8%; grade3, *n*=12, 46.2%) and 39 in the nCT group (grade 0, *n*=4, 10.3%; grade 1, *n*=7, 17.9%; grade 2, *n*=3, 7.7%; grade3, *n*=25, 64.1%) (*P*=0.101). Among the 65 patients, 7 (10.2%) achieved pCR, and 17 (26.2%) achieved MPR. Three (11.5%) of the 26 patients achieved pCR in the nICT group, and four (10.3%) of the 39 patients achieved pCR in the nCT group (*P*=1.0). Six (23.1%) of the 26 patients achieved MPR in the nICT group, and eleven (28.2%) of the 39 patients achieved MPR in the nCT group (*P*=0.645) (Table 2).

**Table 2:** Pathological response in patients who underwent resection

	Category		All patients (*n*=65)	nICT (*n*=26)	nCT (*n*=39)	*P*
ypTNM						0.746
	I		26 (40.0%)	12 (46.2%)	14 (53.8%)	
	II		7 (10.8%)	3 (42.9%)	4 (57.1%)	
	IIIA		8 (12.3%)	4 (50.0%)	4 (50.0%)	
	IIIB		17 (26.2%)	5 (29.4%)	12 (70.6%)	
	IVA		7 (10.8%)	2 (28.6%)	5 (71.4%)	
TRG						0.108
	0		7 (10.8%)	3 (42.9%)	4 (57.1%)	
	1		10 (15.4%)	3 (30.0%)	7 (70.0%)	
	2		11 (16.9%)	8 (72.7%)	3 (27.3%)	
	3		37 (56.9%)	12 (32.4%)	25 (67.6%)	
Pathological response						
	pCR					1.000
		Yes	7 (10.8%)	3 (42.9%)	4 (57.1%)	
		No	58 (89.2%)	23 (39.7%)	35 (60.3%)	
	MPR					0.645
		Yes	17 (26.2%)	6 (35.3%)	11 (64.7%)	
		No	48 (73.8%)	20 (41.7%)	28 (58.3%)	
TNM stage of tumor						0.732
	Down		29 (44.6%)	13 (44.8%)	16 (55.2%)	
	Up		18 (27.7%)	7 (38.9%)	11 (61.1%)	
	Stable		18 (27.7%)	6 (33.3%)	12 (66.7%)	

*nICT* neoadjuvant immunochemotherapy, *nCT* neoadjuvant chemotherapy, *pCR* pathological complete remission, *MPR* major pathological response, *TRG* tumor regression grade

TNM staging was performed again after radical resection of esophageal cancer; 40.0% of the patients were stage I, 10.8% were stage II, 12.3% were stage IIIA, 26.2% were Stage IIIB, and 10.8% were stage IVA, respectively. Downstaging was achieved in 13 (44.8%) patients in the nICT group and 16 (55.2%) patients in the nCT group, respectively (*P*=0.732) (Table 2).

To verify the TRG results, we compared them with MPR and pCR, which showed a significant dependency (*P*< 0.001) (Table 3). Seventeen patients achieved MPR (TRG grade 0, *n*=7, 41.2%; TRG grade 1, *n*=10, 58.8%) (*P*< 0.001), and 7 patients achieved pCR (TRG grade 0, *n*=7, 100%) (*P*< 0.001) after induction of nICT or nCT. In addition, among 17 patients who achieved MPR, 15 (88.2%) achieved tumor downgrading (*P*<0.001). Among 7 patients who achieved pCR, 7 (100%) achieved downgrading (*P*=0.010). This indicated that patients who achieved downstaging showed higher MPR and pCR rates (Table 3).

**Table 3:** Subgroup analyses of MPR and pCR

	MPR (*n*=65)			pCR (*n*=65)		
	Yes (*n*=17)	No (*n*=48)	*P* value	YES (*n*=7)	NO (*n*=58)	*P* value
TRG (grade)			**< 0.001**			**< 0.001**
0	7 (100.0%)	0 (0.0%)		7 (100.0%)	0 (0.0%)	
1	10 (100.0%)	0 (0.0%)		0 (0.0%)	10 (100.0%)	
2	0 (0.0%)	11 (100.0%)		0 (0.0%)	11 (100.0%)	
3	0 (0.0%)	37 (100.0%)		0 (0.0%)	37 (100.0%)	
TNM stage of tumor			**< 0.001**			**0.010**
Down	15 (51.7%)	14 (48.3%)		7 (24.1%)	22 (75.9%)	
UP	2 (11.1%)	16 (88.9%)		0 (0.0%)	18 (100.0%)	
Stable	0 (0.0%)	18 (100.0%)		0 (0.0%)	18 (100.0%)	

*nICT* neoadjuvant immunochemotherapy, *nCT* neoadjuvant chemotherapy, *pCR* pathological complete remission, *MPR* major pathological response, *TRG* tumor regression grade

### Event-free survival (EFS)

**Fig. 1:**
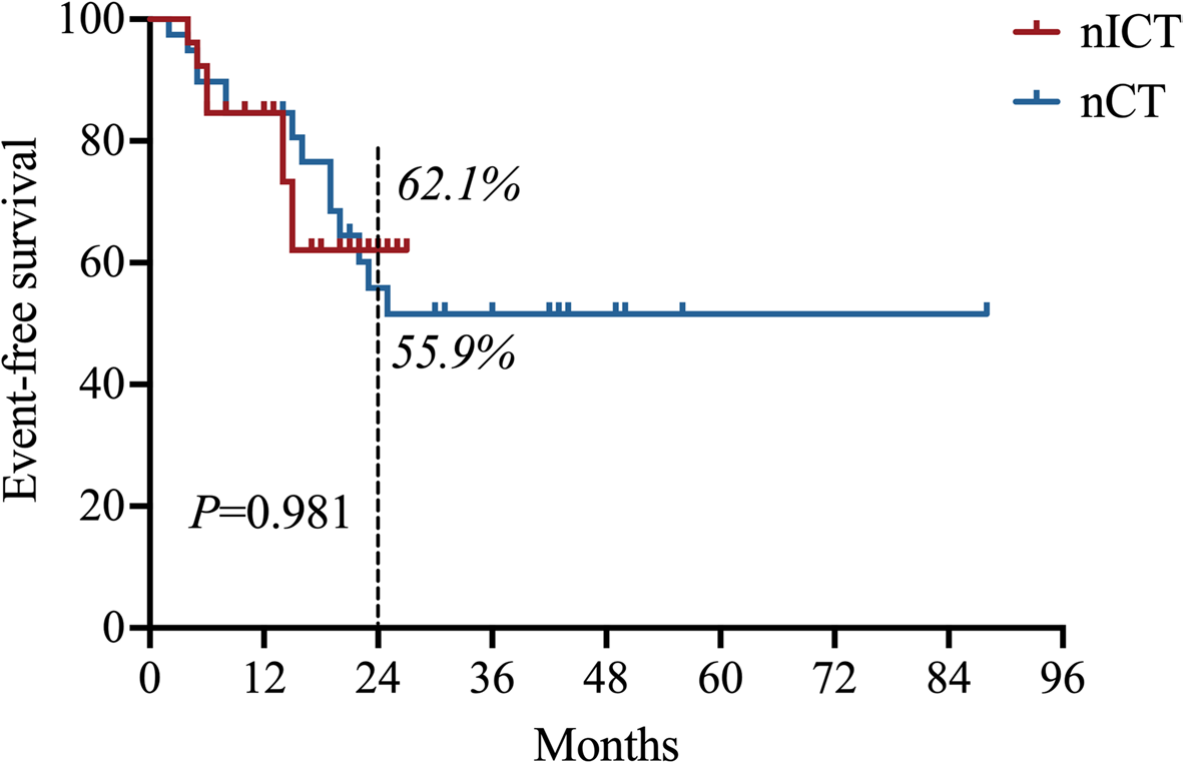
Kaplan-Meier estimates of event-free survival. There was no significant difference in EFS between the nICT and nCT groups (HR=1.011, 95% CI: 0.421–2.425, *P* = 0.981). EFS, event-free survival; nICT, neoadjuvant immunechemotherapy; nCT, neoadjuvant chemotherapy

The median follow-up was 22 months (IQR: 12–36). Postoperative recurrence occurred in 8 patients who received nICT and 14 patients who received nCT alone. The estimated 12-month EFS was 84.6% in both the nICT and nCT groups. The estimated 24-month EFS was 62.1% in the nICT group and 55.9% in the nCT group, respectively. There was no significant difference in EFS between the nICT group and the nCT group (HR=1.011, 95% CI: 0.421–2.425, *P* = 0.981) (Fig. 1).

### Safety

In this study, none of the 65 patients who received nICT or nCT had grade 3 or higher treatment-related adverse events (Table 4). No surgery was postponed due to neoadjuvant treatment-related adverse events. Adverse events related to the protocol treatment occurred in 88.5% of patients in the nICT group and 84.6% in the nCT group. Leukopenia, pruritus, reactive cutaneous capillary endothelial proliferation (RCCEP), and diarrhea were more common in the nICT group (the rates of grade 1 or 2 for these toxicities were 80.8%, 11.5%, 3.8%, and 7.7% in the nICT group and 64.1%, 0%, 0%, and 0% in the nCT group, respectively). Meanwhile, anemia was more frequent in the nCT group (the rate of grade 1 toxicity was 33.3% in the nCT group and 26.9% in the nICT group). Finally, vomit and liver dysfunction were not significantly different between the two groups (the rates of grade 1 for these toxicities were 38.5% and 19.2% in the nICT group and 38.5% and 20.5% in the nCT group, respectively).

**Table 4:** Treatment-related adverse events

	Grade, No. (%)							
	nICT (*n*=26)				nCT (*n*=39)			
	Any	1	2	≥3	Any	1	2	≥3
Treatment-related adverse events	23 (88.5)	17 (65.4)	6 (23.1)	0 (0.0)	33 (84.6)	27 (69.2)	6 (15.4)	0 (0.0)
leukopenia	21 (80.8)	15 (57.7)	6 (23.1)	0 (0.0)	25 (64.1)	18 (46.2)	7 (17.9)	0 (0.0)
Vomit	10 (38.5)	10 (38.5)	0 (0.0)	0 (0.0)	15 (38.5)	15 (38.5)	0 (0.0)	0 (0.0)
Liver dysfunction	5 (19.2)	5 (19.2)	0 (0.0)	0 (0.0)	8 (20.5)	8 (20.5)	0 (0.0)	0 (0.0)
Pruritus	3 (11.5)	2 (7.7)	1 (3.8)	0 (0.0)	0 (0.0)	0 (0.0)	0 (0.0)	0 (0.0)
RCCEP	1 (3.8)	1 (3.8)	0 (0.0)	0 (0.0)	0 (0.0)	0 (0.0)	0 (0.0)	0 (0.0)
Diarrhea	2 (7.7)	2 (7.7)	0 (0.0)	0 (0.0)	0 (0.0)	0 (0.0)	0 (0.0)	0 (0.0)
Anemia	7 (26.9)	7 (26.9)	0 (0.0)	0 (0.0)	13 (33.3)	13 (33.3)	0 (0.0)	0 (0.0)

*nICT* neoadjuvant immunochemotherapy, *nCT* neoadjuvant chemotherapy, *RCCEP* reactive cutaneous capillary endothelial proliferation

## Discussion

In this retrospective study, patients with esophageal cancer who received nICT showed higher pCR and downstaging rates compared with the nCT arm; however, the differences were not statistically significant. Patients who achieved downgrading showed better MPR and pCR rates. Finally, the nICT group did not show significantly better EFS than the nCT group. This suggested that neoadjuvant immunochemotherapy was not superior to chemotherapy alone in esophageal cancer treated with neoadjuvant therapy.

Recently, immunotherapy has become a popular field for treating solid tumors, including esophageal cancer [20]. In multiple Phase II clinical studies, neoadjuvant immunochemotherapy demonstrated safety and efficacy in treating esophageal cancer [12, 14, 21]. And in Phase III clinical study by Sun et al., objective response rates (ORR) were significantly higher in the group treated with pembrolizumab combined with chemotherapy than in the group treated with chemotherapy alone (45.0% vs. 29.3%, *P*<0.001) [22]. At first sight, these observations seemed to be contradictory to our results. However, there were two reasons that can explain why the EFS of the nICT group was not significantly better than the nCT group. Firstly, EFS has been influenced by postoperative adjuvant therapy. Over time, the selection of appropriate postoperative adjuvant therapy may shorten the difference in tumor recurrence or progression time between nICT and nCT groups. It was worth mentioning that further studies were needed to determine whether patients with esophageal cancer needed further adjuvant therapy after surgery. Evidence showed that esophageal cancer patients with residual lymphatic invasion after surgery needed adjuvant therapy [23]. Secondly, some patients with esophageal cancer receiving neoadjuvant therapy may not be sensitive to immunotherapy. It was essential to look for biomarkers that predicted a high response to immunotherapy in patients with esophageal cancer. Unfortunately, this study did not include relevant predictive indicators, including PD-L1. Liu et al. confirmed that ESCC patients with up-regulation of ABCC3, CBR1, and TALDO1 were not sensitive to immunotherapy [24]. In contrast, ESCC patients with enriched immune-related functional pathways (such as NK cells and B cell activity) were sensitive to immunotherapy and had a better prognosis [24].

The prognosis of patients with esophageal cancer receiving neoadjuvant therapy was analyzed according to pathology. Pathological reactions have been used to predict the efficacy of neoadjuvant therapy [17]. Among patients receiving neoadjuvant therapy, those who achieved pCR or MPR had a better prognosis [25, 26]. At present, CT, positron emission tomography/computed tomography (PET-CT), and endoscopic ultrasound (EUS) cannot be adequate to accurately assess pCR in patients with esophageal cancer after neoadjuvant therapy [27]. To a certain extent, the combination of endoscopy and biopsy determined the pathological response of esophageal cancer patients receiving neoadjuvant treatment [28]. In this study, postoperative specimens were used for pathological reaction assessment, 17 patients (TRG 0 or TRG 1) achieved MPR and 7 (TRG 0) pCR. Subgroup analysis confirmed a significant correlation between TRG and pCR/MPR in either the nCT or nICT groups, suggesting that TRG was also a prognostic factor for neoadjuvant therapy. On the other hand, patients who achieved downgrading had higher MPR and pCR rates, and the difference was significant, suggesting that downgrading can also be used as a good prognostic indicator for esophageal cancer patients receiving neoadjuvant therapy and it's consistent with previous research [19].

Other methods have also been found to predict the prognosis of esophageal cancer patients treated with neoadjuvant therapy. It was worth mentioning that metabolic response was superior to histopathology in assessing the prognosis of patients receiving neoadjuvant therapy [29]. A study by Buck et al. proved that using the binary classifier trained on spatial tumor metabolite data for stratification of esophageal adenocarcinoma patients receiving neoadjuvant therapy had an accuracy of 89.7% was better than 70.5% using histopathology [29]. In addition, another study demonstrated that the quantitative response evaluation criteria in solid tumors with multiparametric MRI can assess the prognosis of ESCC patients receiving neoadjuvant therapy [30].

In the future, more clinical trials are needed to confirm our conclusions. ECOG conducted a phase II/III trial to evaluate the efficacy of nivolumab and ipilimumab in perioperative patients (*n*=278) with esophageal adenocarcinoma and gastroesophageal junction adenocarcinoma [3]. The primary endpoints included pCR rates and EFS, which were expected to be completed in 2023 [3]. Yan et al. proposed a phase III clinical trial to further evaluate the role of toripalimab plus chemotherapy in the neoadjuvant setting for patients with resectable ESCC [31]. In addition, improving the efficacy of immunotherapy in patients with esophageal cancer was also the direction of future research. The animal experiment showed that the expression of PD-L1 increased from 45.16 to 77.42% in a dose-dependent manner in a mouse model of esophageal adenocarcinoma induced by chemoradiotherapy (*P*=0.001) [32]. Another study found that trastuzumab can increase tumor PD-L1 expression, and the combination of anti-PD-1 antibodies and trastuzumab can play a synergistic antitumor effect [33]. These suggested that immunotherapy combined with chemoradiotherapy or targeted therapy may bring a higher pathological response rate to patients with esophageal adenocarcinoma.

This study lacked an adequate sample size, and our findings were not statistically significant. The follow-up time was not long enough to see more considerable relapsed events between the nICT and the nCT groups. The retrospective study lacked analyses of markers that predicted the efficacy of neoadjuvant immunotherapy. This study was based on the Chinese population, and the results may not apply to populations in other countries.

## Conclusion

Neoadjuvant PD-1 blockade combined with chemotherapy was not superior to chemotherapy alone for resectable locally advanced esophageal carcinoma patients. However, more studies with long-term follow-up were needed to confirm this result.

## Data Availability

All data generated or analyzed during this study are included in the article. The datasets generated during and/or analyzed during the current study are available from the corresponding author on reasonable request.

## Abbreviations

nCRT: Neoadjuvant chemoradiotherapy
nICT: Neoadjuvant immunochemotherapy
nCT: Neoadjuvant chemotherapy
LAEC: Locally advanced esophageal cancer
PTR: Pathological tumor response
EFS: Event-free survival
pCR: Pathological complete remission
MPR: Major pathological response
PD-1: Programmed death-1
PTR: Pathological tumor response
TRG: Tumor regression grade
CAP: College of American Pathologists
RCCEP: Reactive cutaneous capillary endothelial proliferation
ESCC: Esophageal squamous cell carcinoma
ORR: Objective response rates
PET-CT: Positron emission tomography/computed tomography
EUS: Endoscopic ultrasound
